# A study towards the undirected fluorination and chlorination of cubane 1,4-diester

**DOI:** 10.3762/bjoc.22.101

**Published:** 2026-09-09

**Authors:** Laly Donnier-Valentin, Robin Heinrich, Quentin Fevre-Renault, Julien Legros, Bruno Linclau, Michaël De Paolis

**Affiliations:** 1 Univ Rouen Normandie, INSA Rouen Normandie, Univ Caen Normandie, ENSICAEN, CNRS, Institut CARMeN UMR 6064, 76000 Rouen, France; 2 Ghent University, Department of Organic and Molecular Chemistry, Krijgslaan 281–S4, 9000, Ghent, Belgium

**Keywords:** chlorination, C–H halogenation, cubane 1,4-diester, fluorination, photoactivation

## Abstract

A study towards the undirected C–H fluorination and chlorination of cubane 1,4-diester is described using a user-friendly reagent such as Selectfluor and 1,3-dichloro-5,5-dimethylhydantoin under photoinduction. While the fluorocubane 1,4-diester is formed in low yield (8% yield) due to loss of material, the chlorocubane 1,4-diester was on the other hand prepared in 59% yield without the need for any photocatalyst. Moreover, a proof-of-concept of the chlorination of this strained scaffold was demonstrated using flow technology.

## Introduction

The strained cubane scaffold serves as a three-dimensional bioisosteric alternative to benzene, sharing similar dimensions but lacking π-character [1]. Functionalization of the commercially available cubane 1,4-diester is a critical step to obtain isosteres of trisubstituted benzene rings in molecules of interest. Of note, molecules incorporating the cubane motif often exhibit enhanced solubility, metabolic stability, nonspecific binding, and biological activity compared to their aromatic counterparts [2–5].

**Scheme 1:**
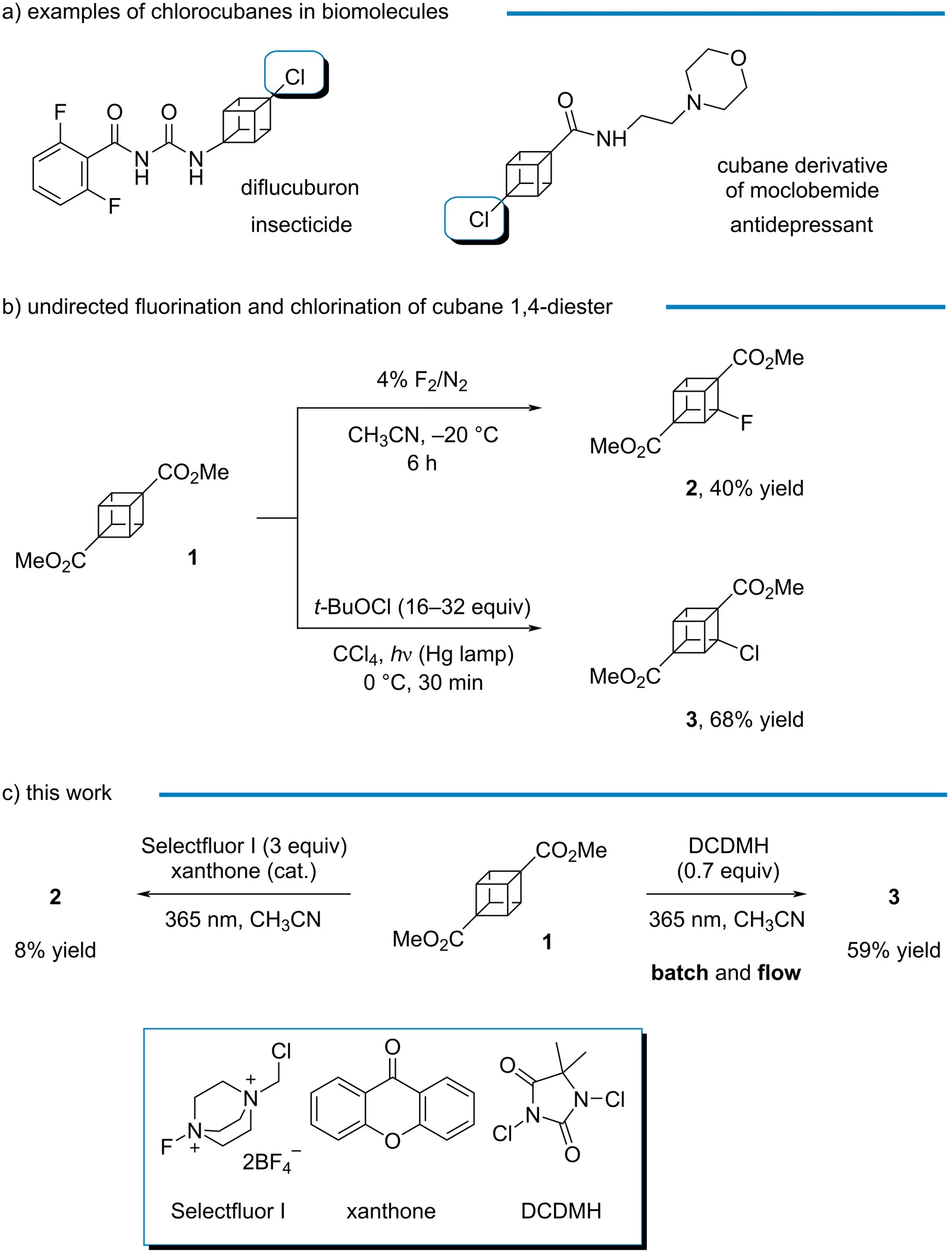
Undirected fluorination and chlorination of cubane 1,4-diester **1**.

Among the functionalization strategies, halogenation – particularly fluorination and chlorination – represents a powerful tool for modulating physicochemical properties of aromatic rings, including acidity, halogen bonding, metabolic stability, and solubility [6–7]. The chlorocubane motif can be found in biomolecules with interesting biological properties like the cubyl analogues of diflubenzuron and of moclobemide (Scheme 1a) [8].

As 1,4-disubstituted halocubane derivatives are prepared by halodecarboxylation, the synthetic routes to 1,2,4-trisubstituted halocubanes are lengthier, requiring prior carboxylation of cubane 1,4-diester [9–10].

As an alternative route, halogenation by hydrogen abstraction of cubane 1,4-diester remains difficult. The presence of ester groups gives rise to strong C–H bonds (103 kcal·mol^−1^) to the framework. While studying the formation of cubyl radicals, Della and Walton observed the deactivation of cubane 1,4-diester toward hydrogen abstraction compared to cubane and alkylcubane [11].

Accordingly, the undirected C–H fluorination or chlorination of cubane 1,4-diester **1** requires harsh conditions (Scheme 1b). The C–H fluorination of **1** was initially reported by Lagodzinskaya in the presence of F_2_ in 40% yield [12]. Elsewhere, Kaleta disclosed the photoinduced C–H chlorination of **1** in 68% yield using an excess of *t*-BuOCl in CCl_4_ [13].

When one aims to selectively monohalogenate cubane 1,4-diester, there is a threefold challenge entailing 1) incomplete conversion, leading to potentially co-eluting mixtures of starting material and halocubane 1,4-diester, 2) polyhalogenation and, 3) degradation of the strained carbon cage.

We wish to describe herein our efforts to perform the C–H fluorination and chlorination of cubane 1,4-diester under user-friendly conditions using readily available reagents and solvents (Scheme 1c).

## Results and Discussion

### Fluorination of cubane 1,4-diester

**Scheme 2:**
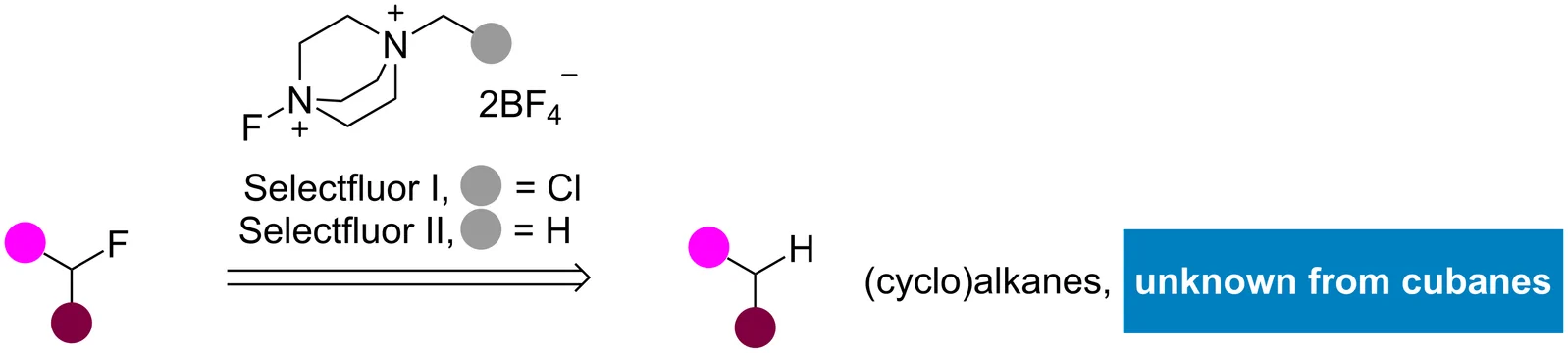
Undirected fluorination of (cyclo)alkanes with Selectfluor reagents.

The study began with the design of a new approach to the fluorocubane 1,4-diester **2** by the undirected fluorination of the commercially available cubane 1,4-diester with Selectfluor instead of fluorine gas. Selectfluor I and II, are practical sources of electrophilic fluorine, employed in undirected C–H fluorination of (cyclo)alkanes [14] involving HAT mechanisms with [15–19] or without photocatalysts [20–22] (Scheme 2).

**Scheme 3:**
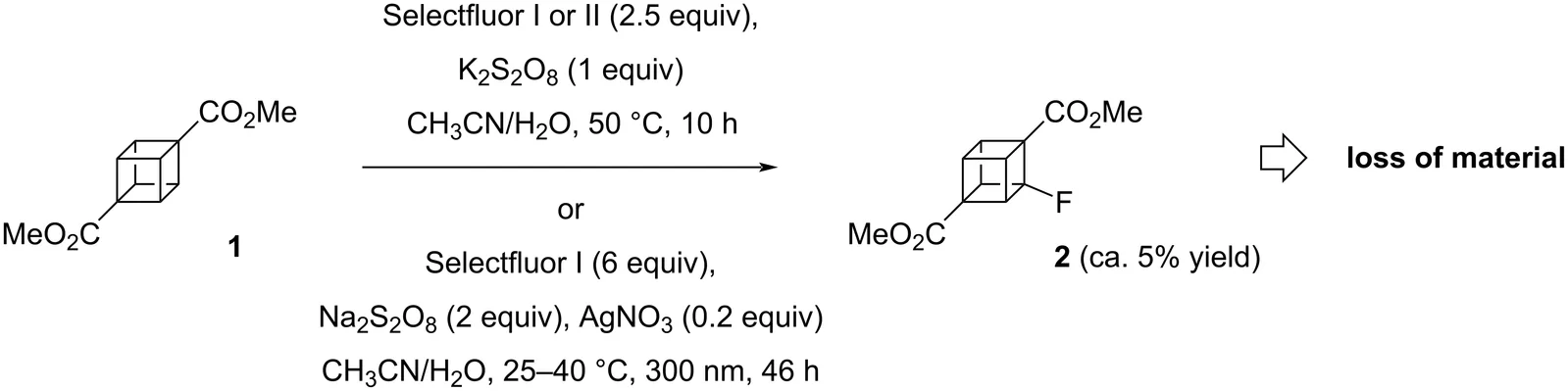
Fluorination of **1** with Selectfluor I or II and peroxydisulfate.

Exposing cubane 1,4-diester to Selectfluor I or II in the presence of peroxydisulfate salts upon heating or under photoactivation led to the isolation of a small amount of fluorocubane **2** (ca. 5% yield) alongside variable amounts of remaining starting material, an important loss of material being noted (Scheme 3).

Slightly more side products were noted in the crude of the reaction with Selectfluor II. Therefore, the first generation of reagent was preferred for the rest of the study. To mitigate degradation, milder conditions were sought using photocatalysis. While attempts to perform the fluorination with tetracyanobenzene, benzophenone, or TBADT were unsuccessful [23], the photocatalyst xanthone (20 mol %) at 300 or 350 nm gave slightly better results (Table 1). In the best scenario, however, **2** was isolated in 8% yield amid partial conversion (ratio **1**:**2** = 1:6). It must be noted that the separation of **2** from the starting material is challenging and achieving higher conversion was detrimental to the mass balance.

**Table 1:** Photoinduced fluorination of **1** and deviations from these conditions.

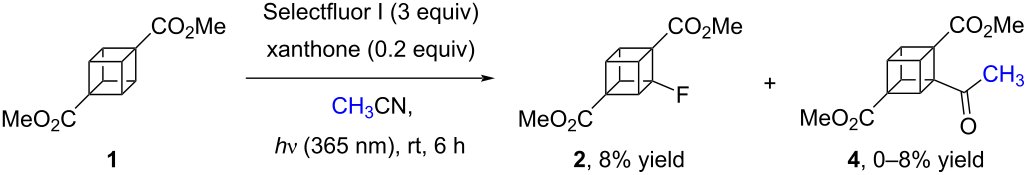			

entry	deviations	**2**	**4**

1^a^	300 nm, CH_3_CN/H_2_O 1:1, 16 h	8%^a^	8%^a^
2^b^	without Selectfluor I, 16 h	–	traces^b^
3	300 nm without Selectfluor I, 16 h	–	not detected^b^
4	Selectfluor I (6 equiv), 85 °C, 16 h without xanthone and *h*ν	traces^b^	not detected^b^
5	Selectfluor I (6 equiv), 300 nm, CH_3_CN/H_2_O 1:1, 24 h without xanthone	traces^b^	traces^b^
6	300 nm in CD_3_CN/H_2_O 1:1, 16 h	8%^a^	**4**-*d*_3_, 5%^a^

^a^Isolated yield; ^b^inspection of the crude reaction by ^1^H NMR spectroscopy (300 MHz).

As a possible explanation of the degradation, we isolated 2-acetylcubane 1,4-diester **4** in variable amounts (up to 8% yield), the highest amount being obtained when operating the C–H fluorination at 300 nm in CH_3_CN/H_2_O (Table 1, entry 1). At this wavelength, the mass balance is lower than at 365 nm. The acetylated product **4** may originate from the reaction of the cubyl radical with acetonitrile followed by hydrolysis of the resulting imine/enamine. Since acetyl, or the parent imine, can be fluorinated with Selectfluor I, it is possible that the fluorination of acetyl **4** or its imine precursor also occurred, followed by degradation. The uncontrolled C–H acetylation of fluorocubane **2** may take place as well. Note that no conversion was observed when operating in methanol instead of acetonitrile (not shown) which is consistent with previous studies on C–H fluorination using the reagent [20–21].

To understand the reaction parameters inducing the formation of **4**, cubane 1,4-diester **1** was exposed to xanthone (0.2 equiv) purposely without Selectfluor I at 365 nm (Table 1, entry 2). Acetyl **4** was observed as traces in the crude of the reaction. The compound was not detected when carrying out the same experiment at 300 nm (Table 1, entry 3).

Furthermore, attempting the C–H fluorination upon heating (Table 1, entry 4, Selectfluor I (6 equiv), CH_3_CN, 85 °C, 16 h) without photocatalyst led to traces of **2** while **4** was not detected. At 300 nm and without Xanthone (Table 1, entry 5), traces of **2** and **4** were observed. Performing the C–H fluorination of **1** in CD_3_CN/H_2_O (Table 1, entry 6, 300 nm) confirmed that the cubane scaffold reacts to a small extend with the solvent since **4**-*d*_3_ was isolated (5% yield) alongside **2** (8% yield).

In summary, the fluorination of cubane 1,4-diester was investigated with Selectfluor I or II with or without a photocatalyst, leading to a small amount (8% yield) of fluorocubane 1,4-diester. As a possible explanation for the low mass balance, the formation of acetyl **4** hints to side reactions with the solvent. This observation motivated us to develop a strategy for the fluorodeiodination of iodocubanes with Selectfluor allowing a directed installation of the fluorine atom [24].

### Chlorination of cubane 1,4-diester

**Scheme 4:**
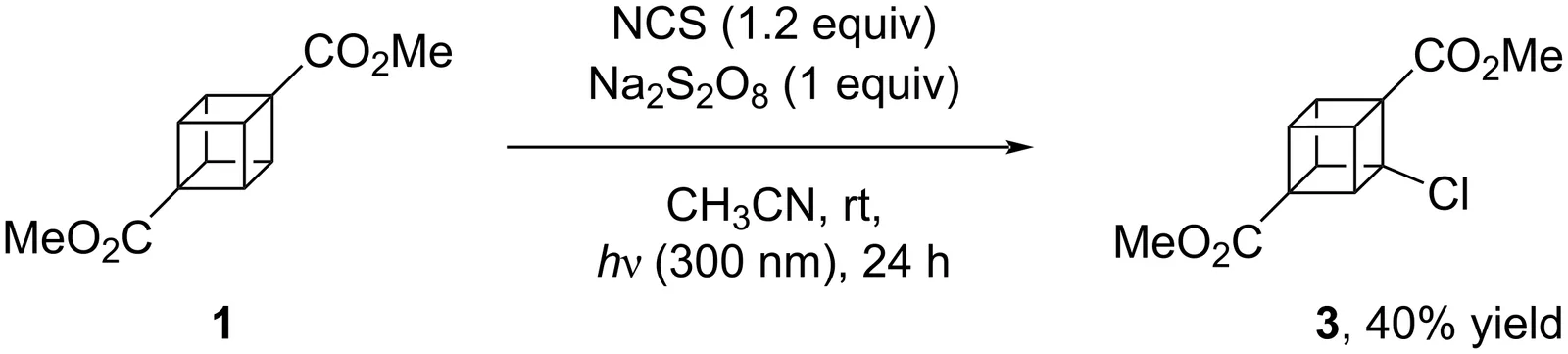
Undirected photoinduced chlorination of **1**.

Pursuing the halogenation of cubane 1,4-diester **1** with mild reagents, we sought to carry out the undirected chlorination of the material. After a screening of conditions, we identified reactions parameters inducing the smooth chlorination of **1**. As detailed in Scheme 4, *N*-chlorosuccinimide (NCS) was combined with photoexcited (300 nm) peroxydisulfate sodium salts to form chlorocubane **3** in 40% yield after 24 h of reaction (ratio **1**/**3** of 1:2.3, estimated by ^1^H NMR spectroscopy).

The contrast with the fluorination of **1** is noteworthy: there was no important loss of material and, probably related, the acetyl product **4** was not detected in the crude of the chlorination reaction.

From this initial result, we carried out an optimization of the reaction parameters (Table 2). Performing the experiment at 365 nm (Table 2, entry 2) was slightly better (44% yield) while increasing the concentration ([**1**] = 0.1 mol/L, Table 2, entry 3) enhanced the ratio **1**/**3** (1:2 to 1:2.6) and yield (52%). On the other hand, a higher concentration (Table 2, entry 4) was unfavorable. Unexpectedly, it was found that the use of sodium peroxydisulfate was slightly detrimental (Table 2, entry 5) and operating without the oxidant led to **3** in 57% yield (ratio **1**/**3** = 1:4.5). Solvent screening revealed that acetonitrile gave a better result than chlorinated solvents or acetone (Table 2, entries 6–9). Eventually, 1,3-dichloro-5,5-dimethylhydantoin (DCDMH) was found to be slightly more efficient than NCS (Table 2, entry 10), giving **3** in 59% yield (ratio **1**/**3** = 1:3.2). The separation of **3** from the starting material is challenging and attempts to achieve higher conversion resulted in negative mass balance.

**Table 2:** Chlorination reaction of **1**.

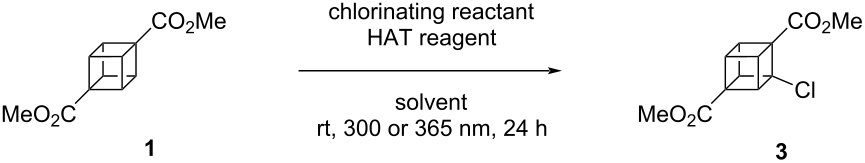						

entry	reactant	HAT reagent (1 equiv)	solvent	[**1**] (mol/L)	ratio **1**/**3**^a^	yield (isolated)

1^b^	NCS^c^	Na_2_S_2_O_8_	CH_3_CN	0.05	1:2.3	40%
2^d^	NCS^c^	Na_2_S_2_O_8_	CH_3_CN	0.05	1:2	44%
3^d^	NCS^c^	Na_2_S_2_O_8_	CH_3_CN	0.1	1:2.6	52%
4^d^	NCS^c^	Na_2_S_2_O_8_	CH_3_CN	0.2	1:1.9	48%
5^d^	NCS^c^	–	CH_3_CN	0.1	1:4.5	57%
6^d^	NCS^c^	–	CH_2_Cl_2_	0.1	1:1.2	nd
7^d^	NCS^c^	–	acetone	0.1	nr	
8^d^	NCS^c^	–	DCE	0.1	nr	
9^d^	NCS^c^	–	CHCl_3_	0.1	nr	
**10** ^d^	DCDMH^e^	–	CH_3_CN	0.1	1:3.2	59%

^a^Estimated by ^1^H NMR spectroscopy. ^b^300 nm; ^c^1.2 equiv; ^d^365 nm; ^e^0.7 equiv.; nr: no reaction; nd: not determined; DCE: 1,2-dichloroethane.

We next sought to develop the chlorination reaction in flow conditions (in a Vapourtec^®^ R-series) as a proof-of-concept with aims both to (i) scale up and (ii) to evaluate the impact of the *t*_R_ (residence time) on the amount of polychlorocubane products (Table 3). In batch, small amounts of polychlorocubanes were detected and we were curious to examine whether these products could be suppressed.

**Table 3:** Chlorination reaction of **1** in flow.

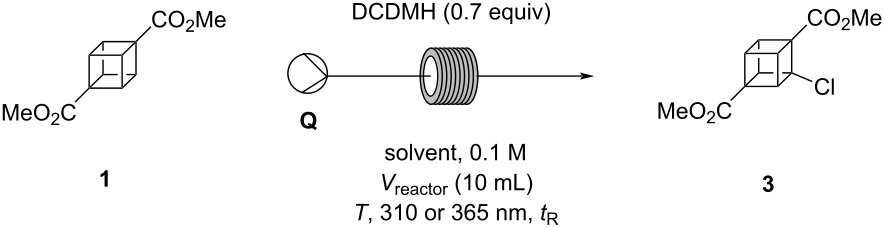					

entry	flow rate **Q** (mL/min)	solvent	*T* (°C)	*t*_R_ (min)	ratio **1**/**3**^a^

1^b,c^	0.4	CH_3_CN	28	25	–
2^b,c^	0.4	CH_3_CN/CH_2_Cl_2_ 10:1	28	25	1:0.42
3^b,c^	0.4	CH_3_CN/CH_2_Cl_2_ 4:1	28	25	1:0.82
4^c,d^	0.22	CH_3_CN	30	45	1:1.32
5^d,e^	0.33	CH_3_CN	28	30	1:1.13

^a^Estimated by ^1^H NMR spectroscopy; ^b^partial precipitation; ^c^365 nm; ^d^heating at 30 °C and sonication of the solution of **1** (*c* = 0.1 M); ^e^310 nm.

It was noted that when using CH_3_CN as solvent, the substrate **1** partially precipitated in the tube (flow rate 0.4 mL/min, Table 3, entry 1). Therefore, a co-solvent CH_2_Cl_2_ was introduced (Table 3, entries 2 and 3) and even though partial solubilization of **1** was achieved, low amounts of **3** were formed. After 45 min of residence, chlorocubane **3** became the major product (ratio **1**/**3** = 1:1.32) when using a pre-heated and sonicated initial solution of **1** in CH_3_CN (Table 3, entry 4). However, small amounts of polychlorinated cubanes were detected. A similar setting was tested at 310 nm (Table 3, entry 5) which led to the isolation of **3** in 41% yield (61% based on remaining starting material).

**Scheme 5:**
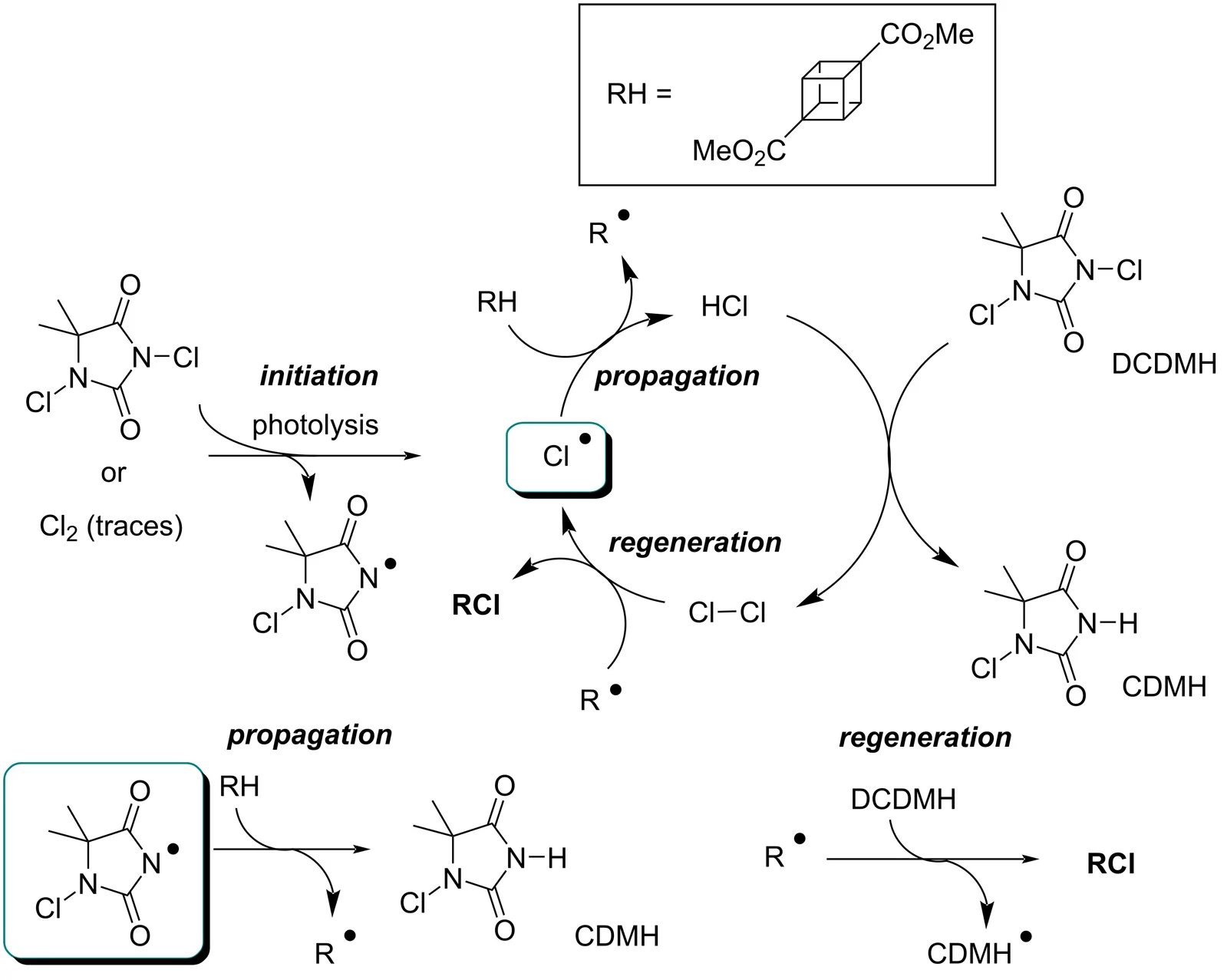
Proposed mechanism.

While the chlorination of **1** was achieved by Kaleta using a large excess of *t*-BuOCl (16–32 equiv) under photoactivation [13], the utility of the current method involving DCDMH (0.7 equiv) is noteworthy. Since the photolysis of NCS has been described by MacMillan at 450 nm to release chlorine radicals (Cl^•^) [25], a tentative mechanism proposal would imply that N-centered and chlorine radicals are similarly released by photolysis of DCDMH or of molecular chlorine contained as traces in the reagent (Scheme 5). When the experiment was conducted in the dark, no chlorination occurred. Propagating the chain, the radicals will in turn engage in cubane HAT to give HCl (BDE = 102 kcal·mol^−1^), a process observed in other contexts [26].

Note that HAT activation of cubane is a challenging transformation (BDE = 103 kcal·mol^−1^) performed here with stable and available reagents DCDMH or NCS. The generation of cubyl radicals from cubane 1,4-diester traditionally requires photoexcited strong and unstable oxidants, such as *t*-BuOX (X = Cl [13], I [27]), or tetra-*n*-butylammonium decatungstate (TBADT) [28–33].

The reaction of HCl with DCDMH would generate molecular chlorine [34], which by reaction with cubyl radicals may subsequently form chlorocubane and regenerate Cl^•^. An alternative and parallel pathway may involve the reactivity of the N-centered radical chlorodimethylhydantoin (CDMH) [35], which could form the cubyl radical by HAT and regeneration of the N-centered radical species (CDMH^•^) after its reaction with DCDMH [36].

## Conclusion

Mild conditions were developed to perform undirected fluorination and chlorination of the commercially available cubane 1,4-diester, which will be of interest for the synthesis of substituted benzene isosteres. Whilst the fluorination protocol was limited in efficiency compared to the fluorodeiodination strategy, user-friendly reagents and solvents were successfully employed for the chlorination reaction. The transformation was performed in batch and we demonstrated a proof-of-concept flow implementation. These advances should facilitate the incorporation of chlorocubyl motifs into molecules in various fields of investigation spanning pharmaceuticals, functional materials, and crop science.

## Supporting Information

File 1 Experimental section and copies of spectra.

## Data Availability

All data that supports the findings of this study is available in the published article and/or the supporting information of this article.
