# Supplementary material for: A study towards the undirected fluorination and chlorination of cubane 1,4-diester
Source: Beilstein J Org Chem. 2026 Sep 9;22:1246–53. doi: 10.3762/bjoc.22.101 (PMC13575343; doi:10.3762/bjoc.22.101)

BEILSTEIN JOURNAL OF ORGANIC CHEMISTRY

Supporting Information

for

A study towards the undirected fluorination and chlorination of cubane 1,4-diester

Laly Donnier-Valentin, Robin Heinrich, Quentin Fevre-Renault, Julien Legros, Bruno Linclau and Michaël De Paolis

Beilstein J. Org. Chem. **2026,** *22,* 1246–1253. doi:10.3762/bjoc.22.101

Experimental section and copies of spectra

License and Terms: This is a supporting information file under the terms of the Creative Commons Attribution License (https://creativecommons.org/licenses/by/4.0). Please note that the reuse, redistribution and reproduction in particular requires that the author(s) and source are credited and that individual graphics may be subject to special legal provisions.
The license is subject to the *Beilstein Journal of Organic Chemistry* terms and conditions: (https://www.beilstein-journals.org/bjoc/terms)

General Information S2

Synthesis of dimethyl 2-fluorocubane-1,4-dicarboxylate (**2**) S3

Dimethyl 2-acetylcubane-1,4-dicarboxylate **4** and **4**-$\boldsymbol{d_3}$ S6

Synthesis of dimethyl 2-chlorocubane-1,4-dicarboxylate (**3**) S11

General Information

^{1}H NMR (300 MHz) and ^{1}H NMR (400 MHz) spectra were recorded respectively on Bruker Avance III 300 MHz and Bruker Ascend EVO 400 in deuterated chloroform $CDCl_3$ and are internally referenced to residual protic $CDCl_3$ (δ 7.26 ppm). ^{13}C NMR (75 MHz) and ^{13}C NMR (101 MHz) spectra were recorded respectively on Bruker Avance III 300 MHz and Bruker Ascend EVO 400 in deuterated chloroform $CDCl_3$ and are internally referenced to residual protic $CDCl_3$ (δ 77.16 ppm). ^{19}F NMR (282 MHz) spectra were recorded on Bruker Avance III 300 MHz without reference.
Chemical shifts (δ) are given in parts per million (ppm) and coupling constants (*J)* are given in Hertz.

High resolution mass spectra were acquired on Waters LCT Premier apparatus coupled with LC Waters Aquity device or on JEOL AccuTof 4G apparatus coupled with a GC HP Agilent 7890 device. High-resolution mass data were recorded on Q-TOF instrument with an electrospray source in API, EI or ESI mode. Infrared spectra were recorded on Perkin Elmer ATR universal sampler 100 spectrum.

Visualization of thin-layer chromatography (TLC) plates was accomplished with UV (254 nm) and ammonium molybdate staining solution (12 g of ammonium molybdate tetrahydrate, 15 mL of 95% H_2SO_4, 235 mL of distilled water).
Selectfluor I and II, and DCDMH were purchased from Fisher, ABCR and Sigma Aldrich.
Cubane 1,4-diester was either prepared according to known method or purchased from ABCR.
Photochemical reactions performed at 365 nm were realized in borosilicate tube in a EvoluChem photochemical device (PhotoRedOx Box™ from EvoluChem equipped with a EvoluChem LED 365PF blue lamp and a built-in fan, with no filter and placed on a stirring plate). Flow study was performed on Vapourtec® R-Series equipped with 310 and 365 nm lamp.
Experiments at 300 nm were carried out with Rayonet photochemical device (RPR-200 Photochemical Reactor) from Rayonet equipped with ten RPR-3000Å UV blue lamps.
Unless otherwise indicated, technical grade MeCN was employed for the experiments of C-H fluorination and chlorination.

Synthesis of dimethyl 2-fluorocubane-1,4-dicarboxylate (2)

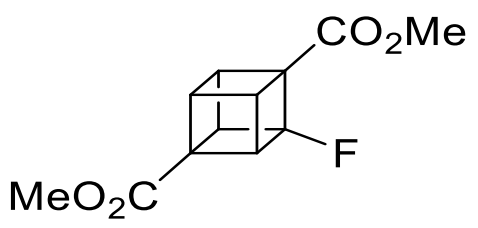

With sodium persulfate and silver nitrate:

To a dried quartz tube glassware, equipped with a magnetic stir bar, was added dimethyl 1,4-cubanedicarboxylate **1** (200 mg, 0.908 mmol, 1 equiv.), silver nitrate (30.9 mg, 0.182 mmol, 0.2 equiv.), Selectfluor I (1.93 g, 5.45 mmol, 6 equiv.) and sodium persulfate (432 mg, 1.82 mmol, 2 equiv.). The tube was degassed with an argon flow then degassed acetonitrile (10 mL) was added followed by degassed water (10 mL). The solution was stirred and irradiated 300 nm in a Rayonet photochemical device (RPR-200 Photochemical Reactor from Rayonet equipped with ten RPR-3000Å UV blue lamps, with a fan and a stirring plate at the bottom, with no filter and the tube placed at approximately 10 cm from the lamps) during 46 h at a temperature between 25 and 40°C. After the irradiation, the crude reaction was extracted with diethyl ether and the organic layer was washed with a saturated aqueous solution of sodium thiosulfate. Then, the solution was dried with $MgSO_4$, filtered and the solvent was concentrated in vacuo. Purification was performed on flash chromatography (eluted with cyclohexane/ethyl acetate/dichloromethane from 100/0/0 to 85/5/10). The pure product **2** was isolated as colorless crystals (6 mg, 3 % yield). Another fraction containing **2** along with the starting material was also collected (20 mg, ^{1}H NMR ratio **1**:**2** = 28:62).

With xanthone:

Dimethyl 1,4-cubanedicarboxylate **1** (50 mg, 0.227 mmol, 1 equiv), xanthone (8.9 mg, 0.2 equiv) and Selectfluor I (254 mg, 0.717 mmol, 3 equiv) were placed in a flask. It was purged with an argon flow then degassed acetonitrile (1 mL) were added. The reaction mixture was inserted into a vial (4 mL volume) or a quartz tube and the reactor was irradiated at rt under 365 nm overnight.

The solution was diluted with Et_2O (10 mL) and the suspension was filtrated. The solvent was removed to give a residue (~60 mg). Purification was performed by column chromatography with eluant cyclohexane/AcOEt (80/20) to give 5 mg (8 %) of 2-fluorocubane 1,4-diester **2**.

^{1}H NMR (300 MHz, $CDCl_3$) δ 4.62 – 4.48 (m, 2H), 4.11 – 4.00 (m, 3H), 3.69 (s, 3H), 3.66 (s, 3H); **^{13}C NMR** (75 MHz, $CDCl_3$) δ 170.8 (d, *J* = 2.3 Hz), 168.3, 99.3 (d, *J* = 338.4 Hz), 65.1 (d, *J* = 26.7 Hz), 57.3 (2C, d, *J* = 25.9 Hz), 52.06, 52.06, 49.7 (d, *J* = 3.9 Hz), 48.9 (d, *J* = 11.1 Hz), 41.0 (2C, d, *J* = 3.1 Hz); **^{19}F NMR** (282 MHz, $CDCl_3$) δ -145.79 (ddt, *J* = 15.7, 9.6, 6.0 Hz); **HRMS** (EI) *m/z:* $[M]^{+\cdot}$ Calcd for $C_{12}H_{11}FO_4$ 238.0641; Found 238.0636.

^{1}H NMR (300 MHz, $CDCl_3$)

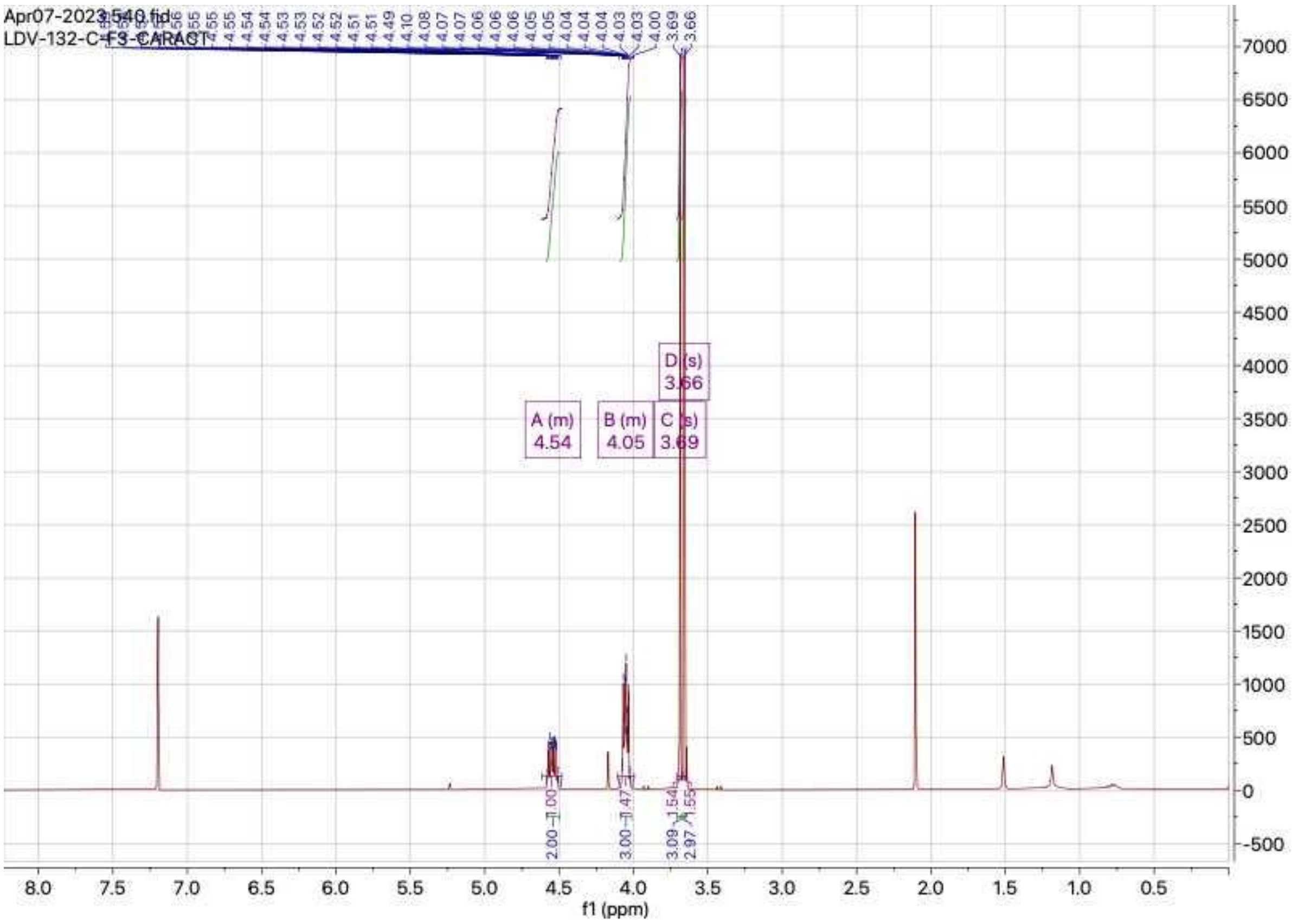

^{13}C NMR (75 MHz, $CDCl_3$)

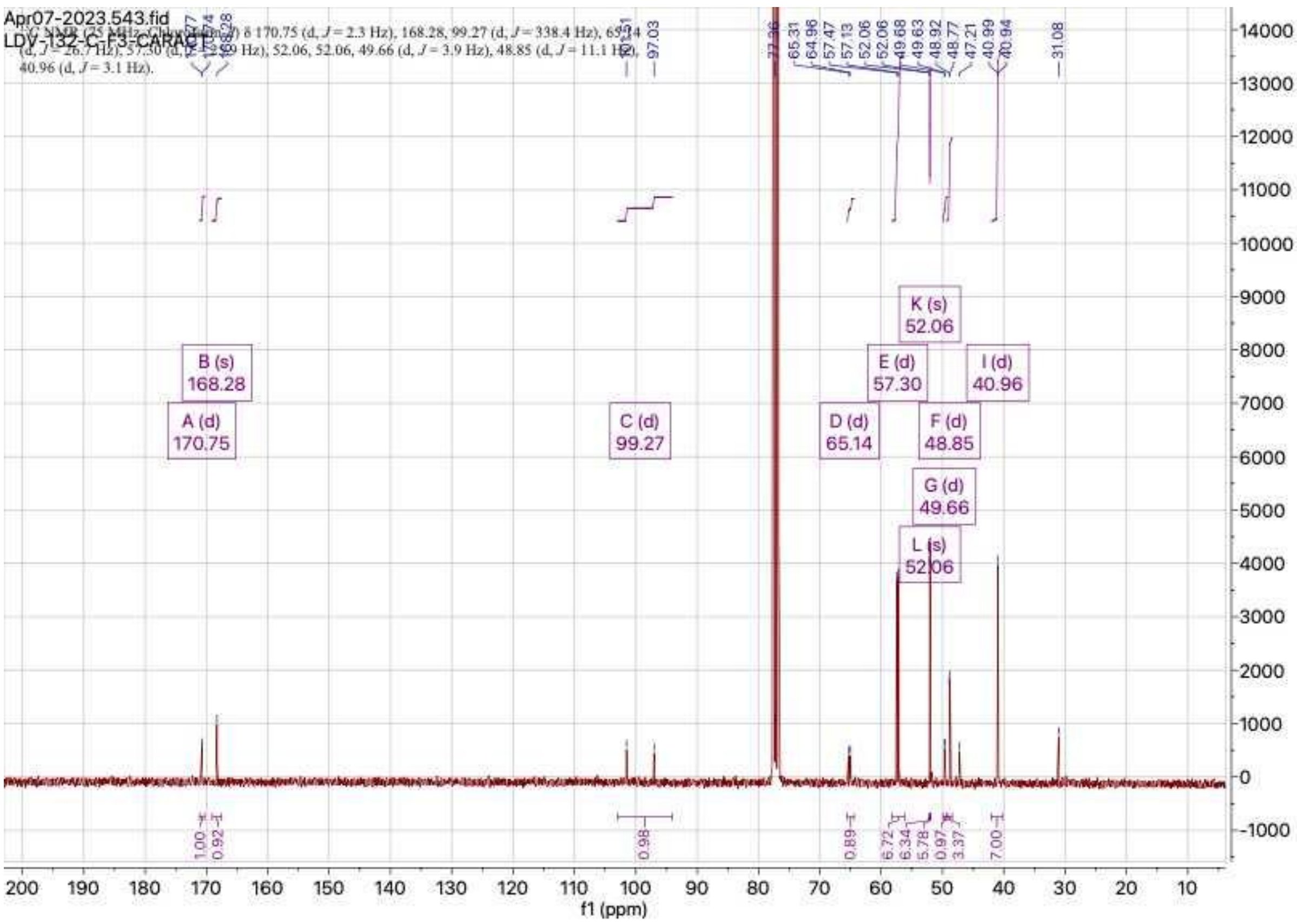

^{13}C NMR (75 MHz, $CDCl_3$)

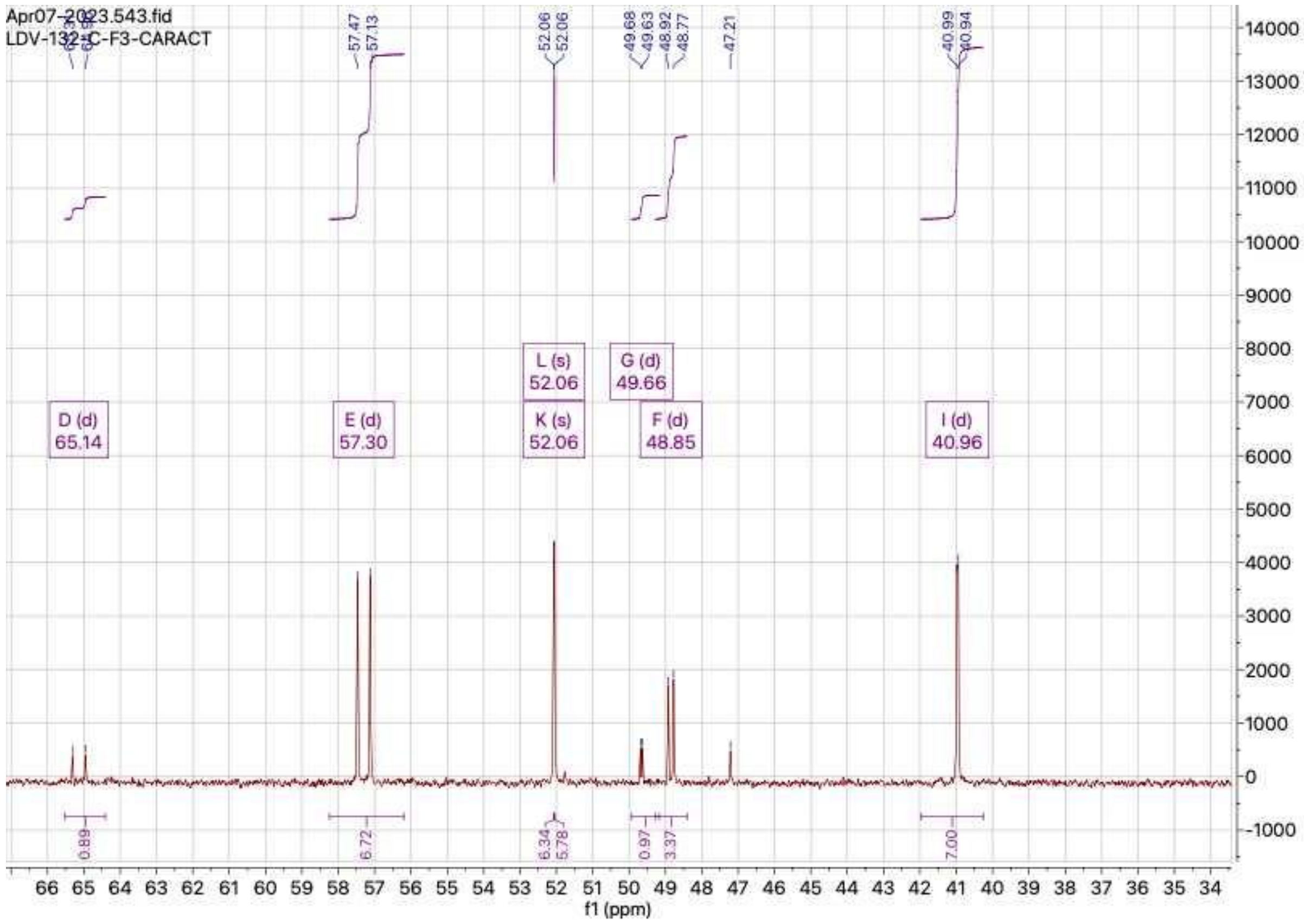

^{19}F NMR (282 MHz, $CDCl_3$)

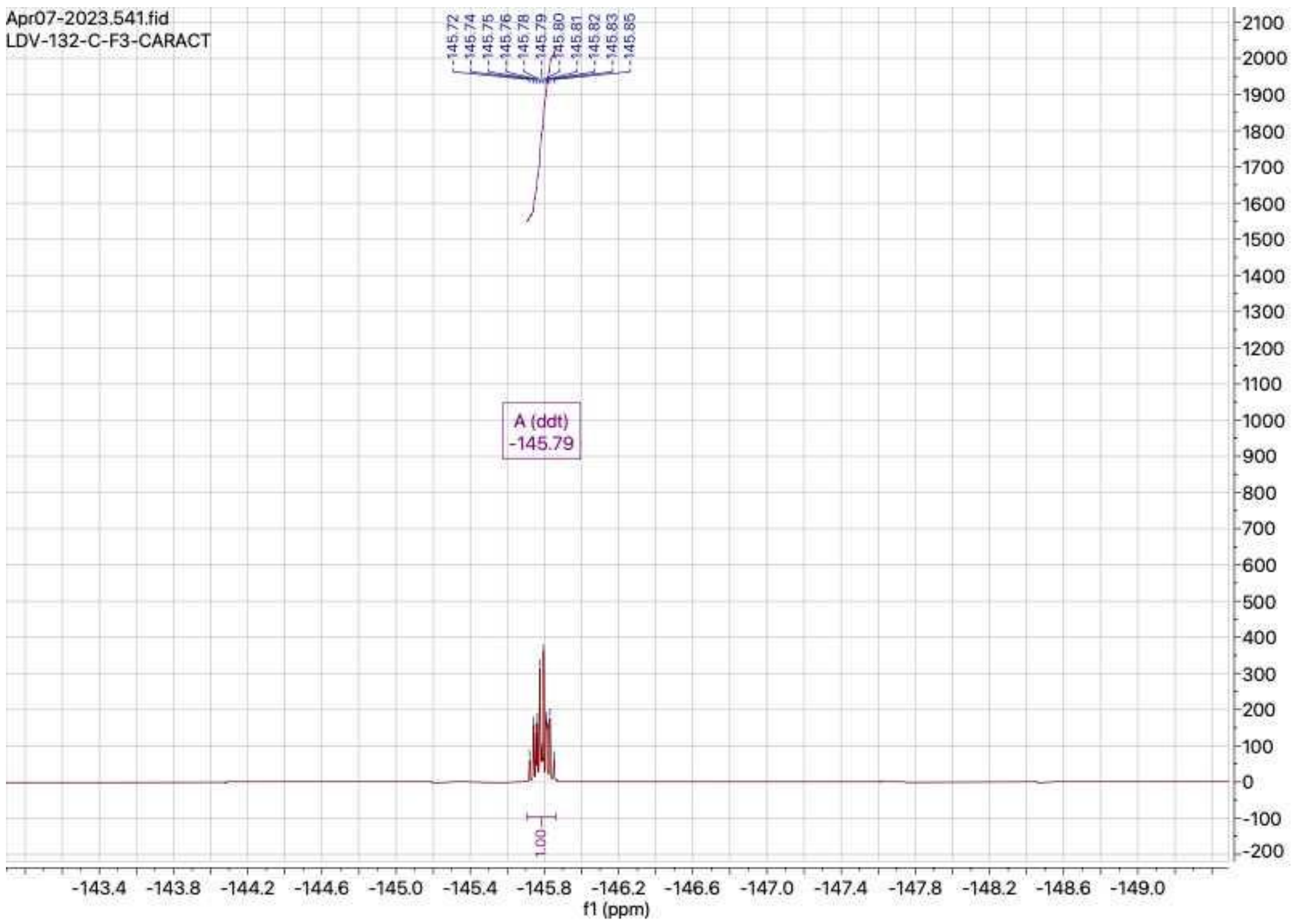

Synthesis of dimethyl 2-acetylcubane-1,4-dicarboxylate (4) and 4-*d*$_3$

Dimethyl 2-acetylcubane-1,4-dicarboxylate (4)

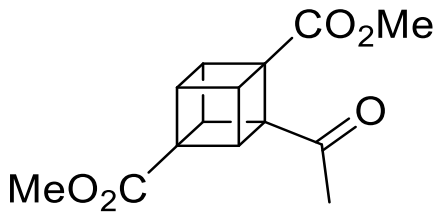

Isolated as a side product (up to 8% yield) during the fluorination experiments with Selecfluor. Dimethyl 1,4-cubanedicarboxylate **1** (50 mg, 0.227 mmol, 1 equiv), xanthone (8.9 mg, 0.2 equiv) and Selectfluor I (254 mg, 0.717 mmol, 3 equiv) were placed in a flask. It was purged with an argon flow then degassed acetonitrile (2 mL) and H_2O (2 mL) were added. The reaction mixture was inserted into a quartz tube and the reactor was irradiated at rt under 300 nm overnight in a Rayonet device.

The solution was diluted with Et_2O (10 mL) and the suspension was filtrated. The solvent was removed to give a residue (~15 mg). Purification was performed by column chromatography with eluant cyclohexane/AcOEt (80/20) to give 3 mg (8 %) of **4**.

^{1}H NMR (400 MHz, $CDCl_3$) δ 4.49 – 4.47 (m, 2H), 4.23 – 4.20 (m, 3H), 3.72 (s, 6H), 2.16 (s, 3H); **^{13}C NMR** (100 MHz, $CDCl_3$) δ 202.9, 169.0 (2C), 62.4, 53.0 (2Cq), 52.1, 52.0, 49.4 (2C), 47.8, 44.9 (2C), 26.1; **HRMS** (API) *m/z:* $[M]^+$ Calcd for $C_{14}H_{14}O_5$ 262.08412; Found 262.08349.

^{1}H NMR (400 MHz, $CDCl_3$)

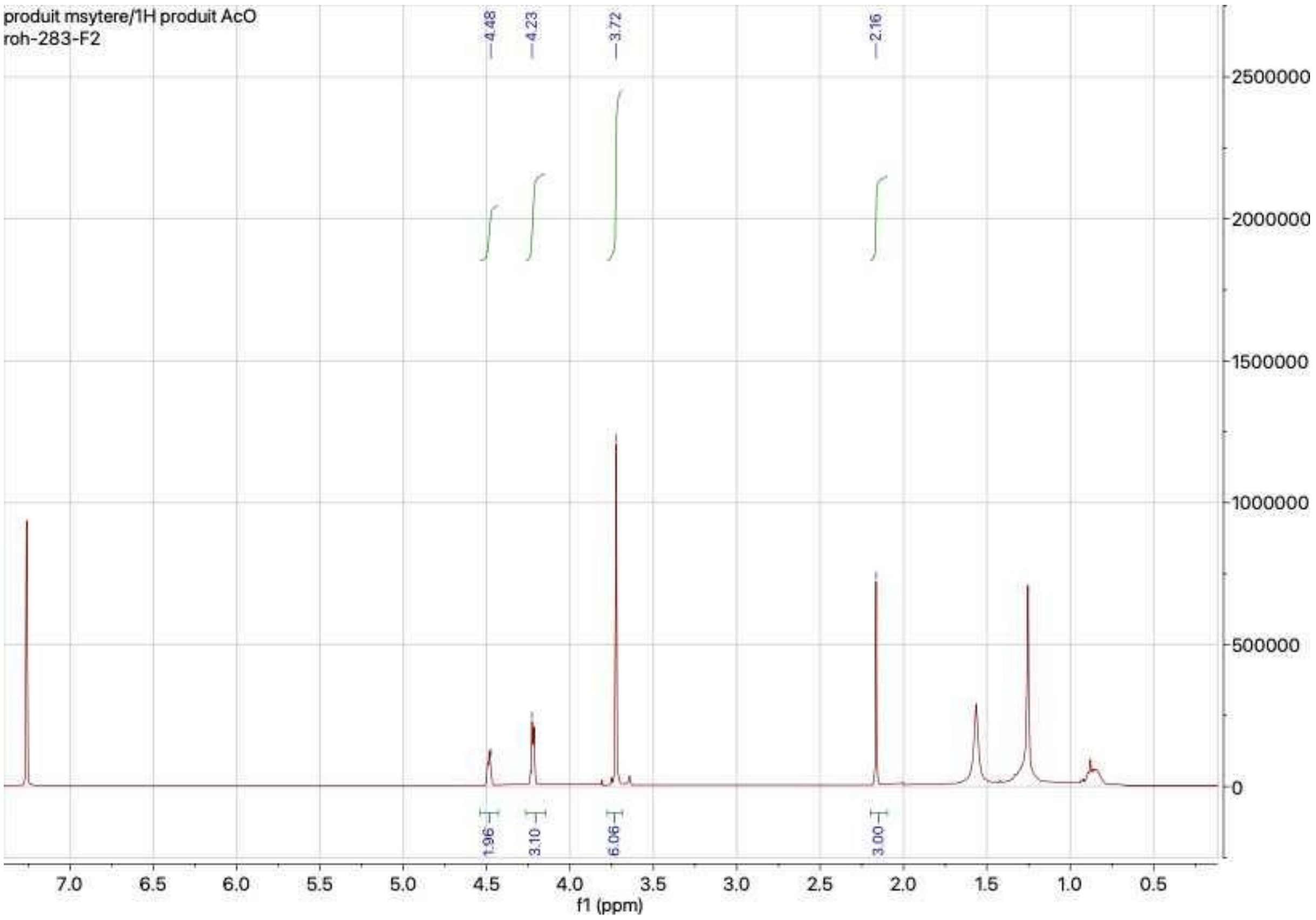

^{13}C NMR DEPT (101 MHz, $CDCl_3$)

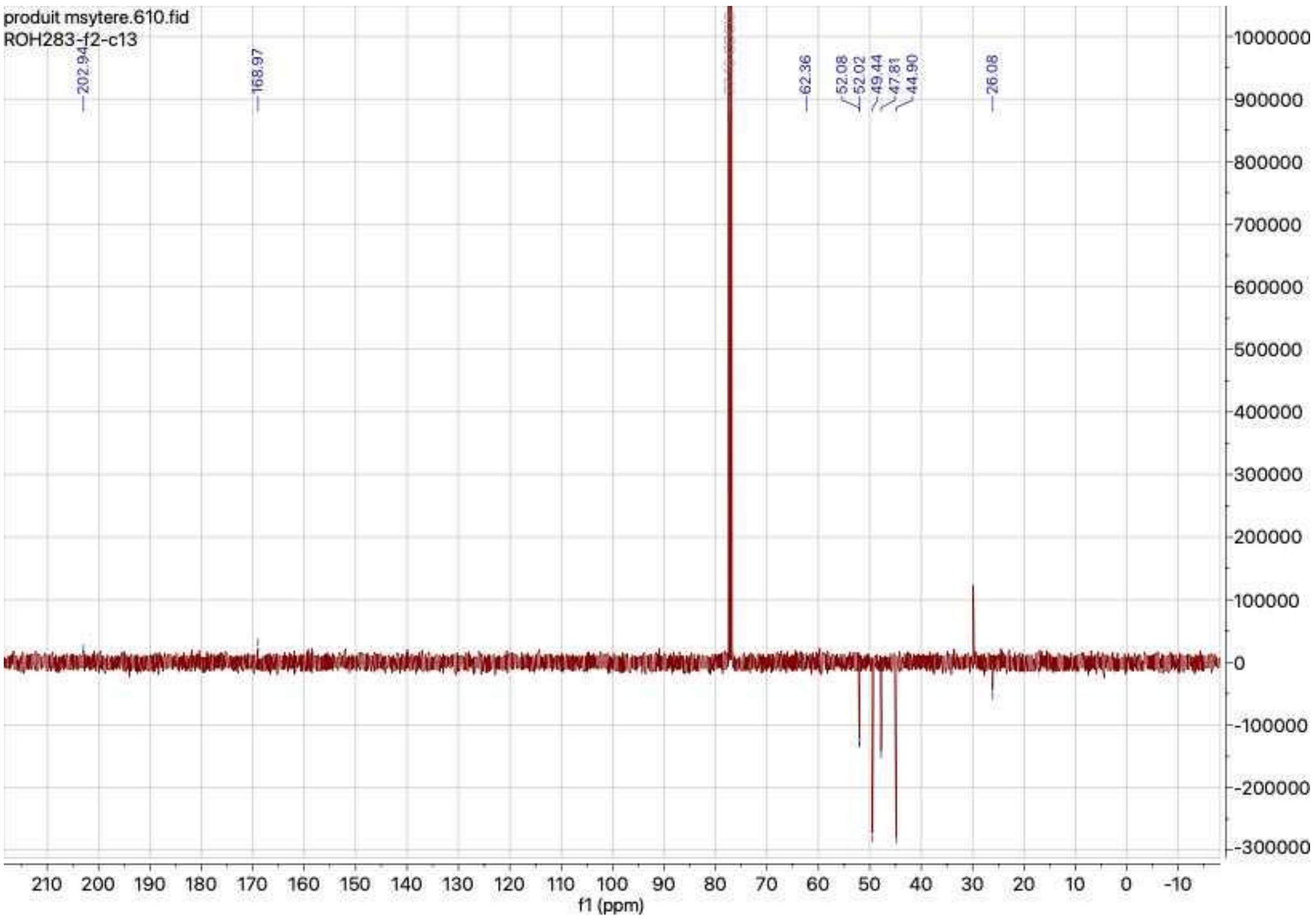

HSQC (400 MHz, $CDCl_3$)

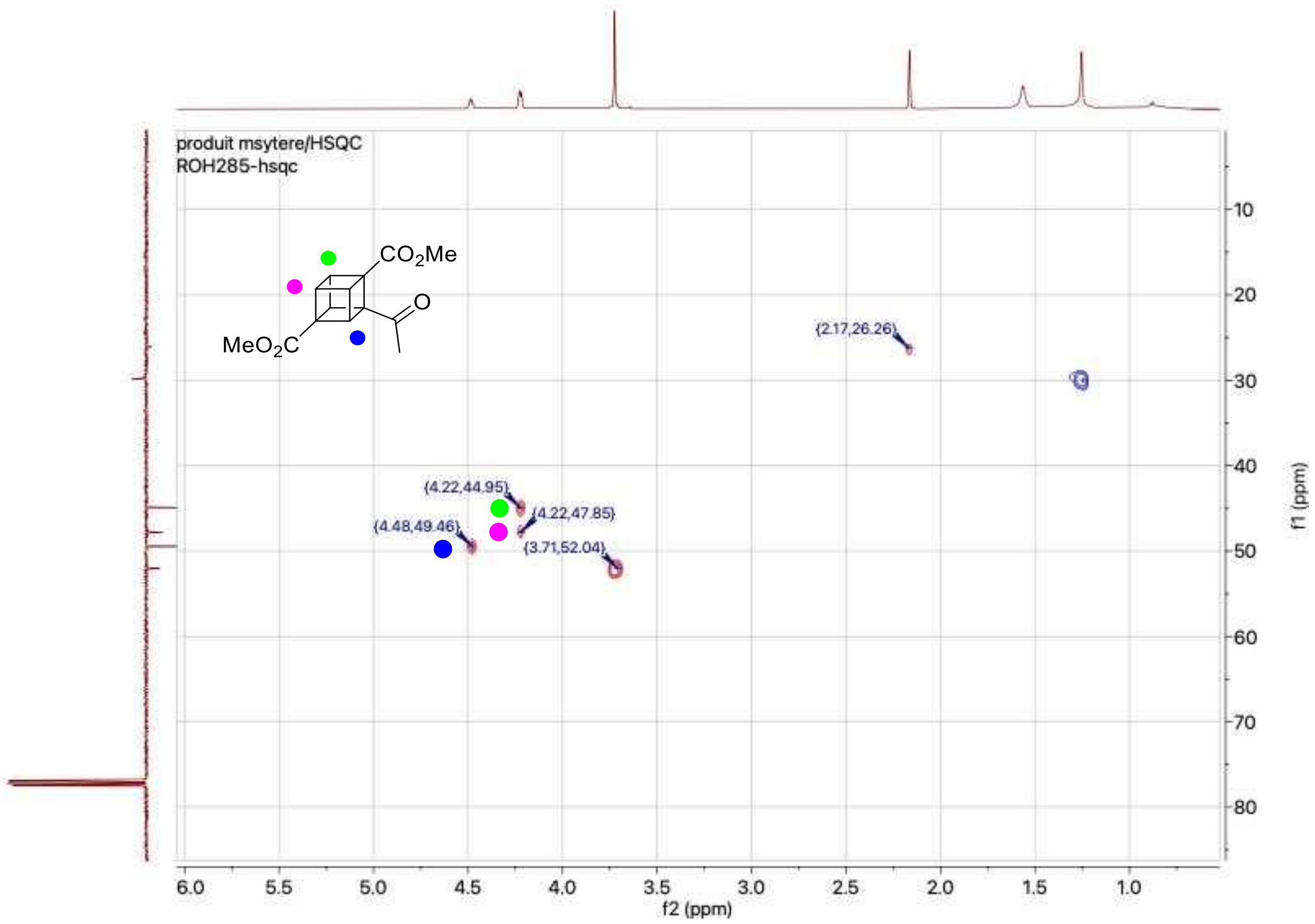

HMBC (400 MHz, $CDCl_3$)

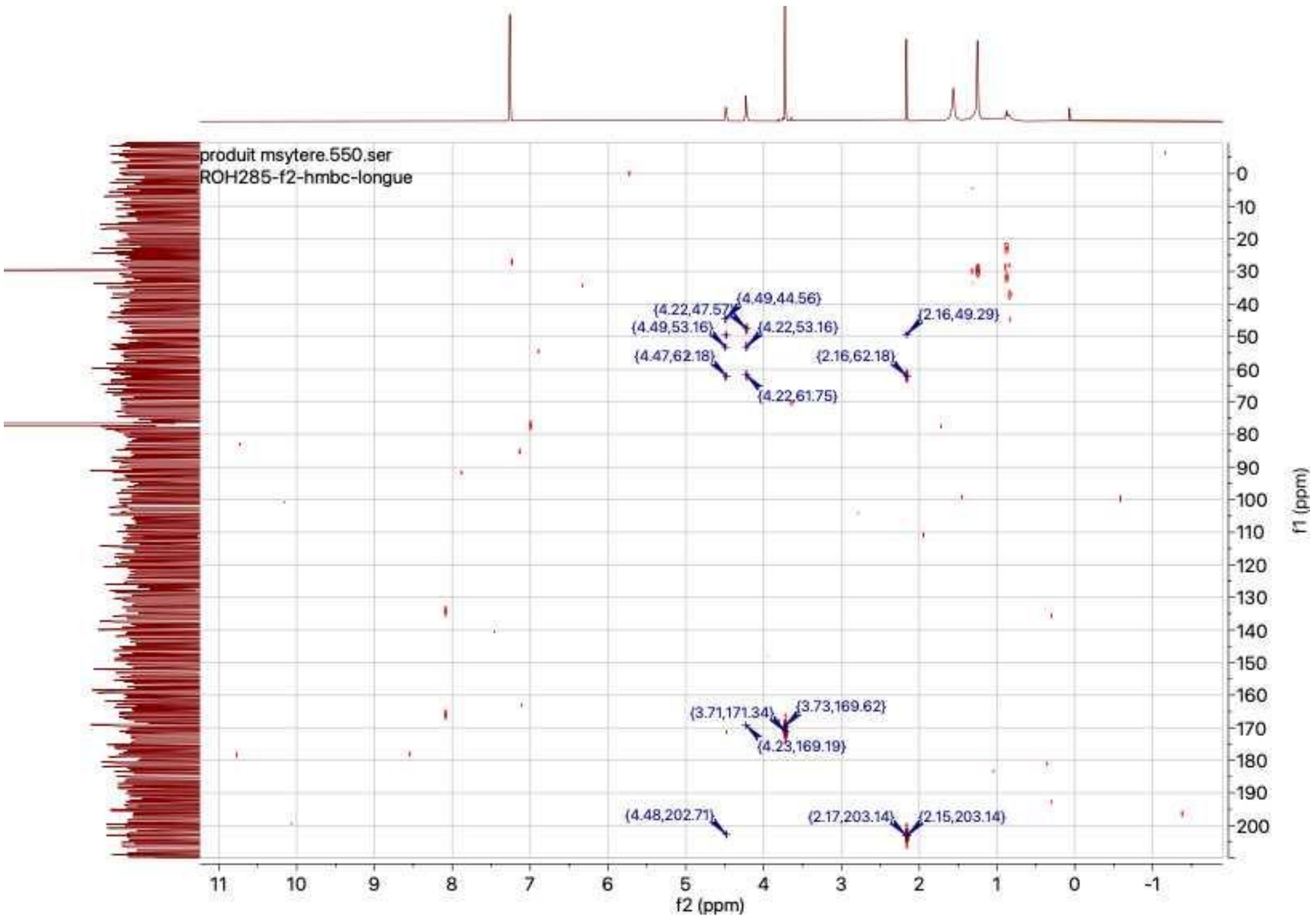

HMBC (400 MHz, $CDCl_3$) zoom 1

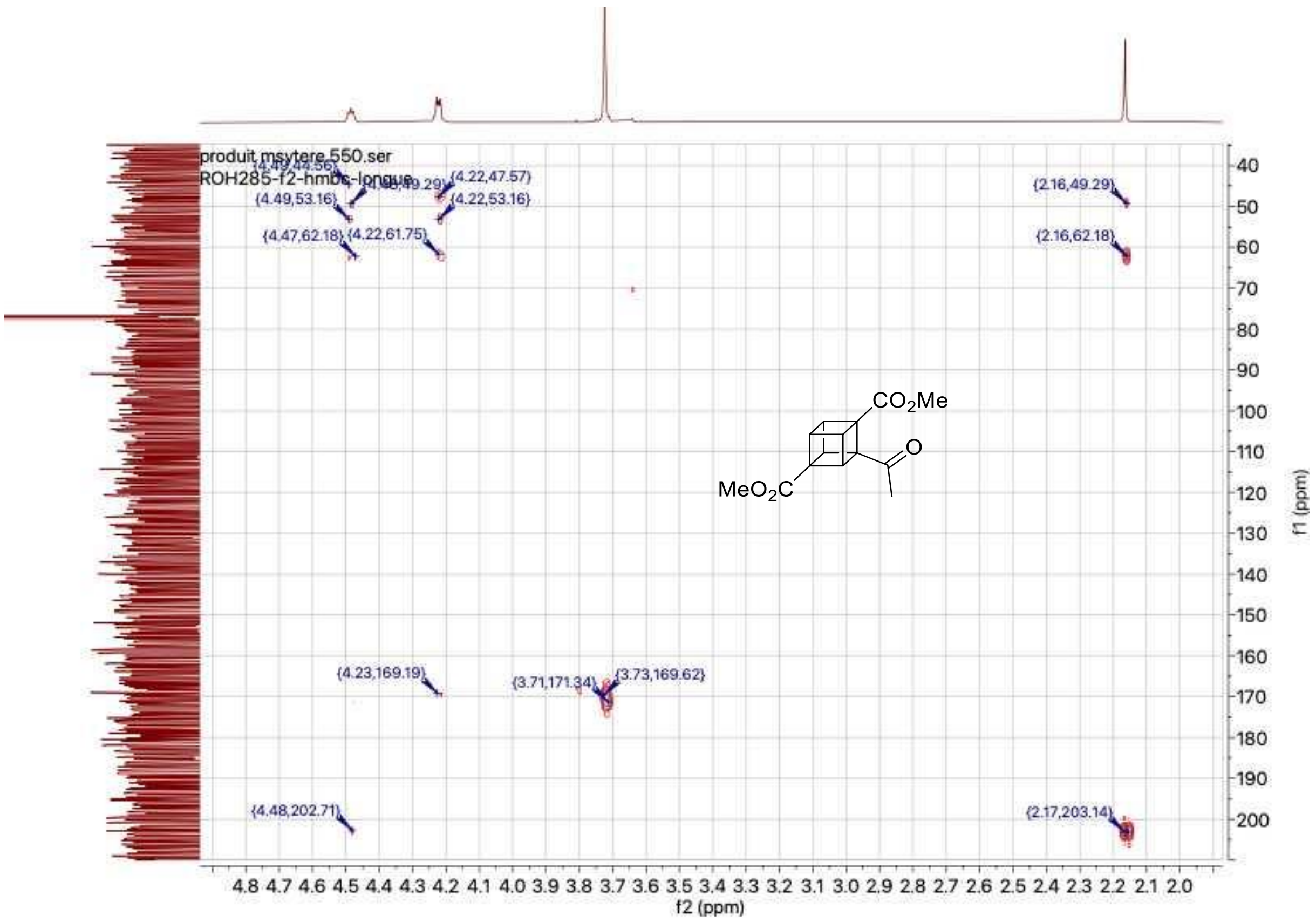

HMBC (400 MHz, $CDCl_3$) zoom 2

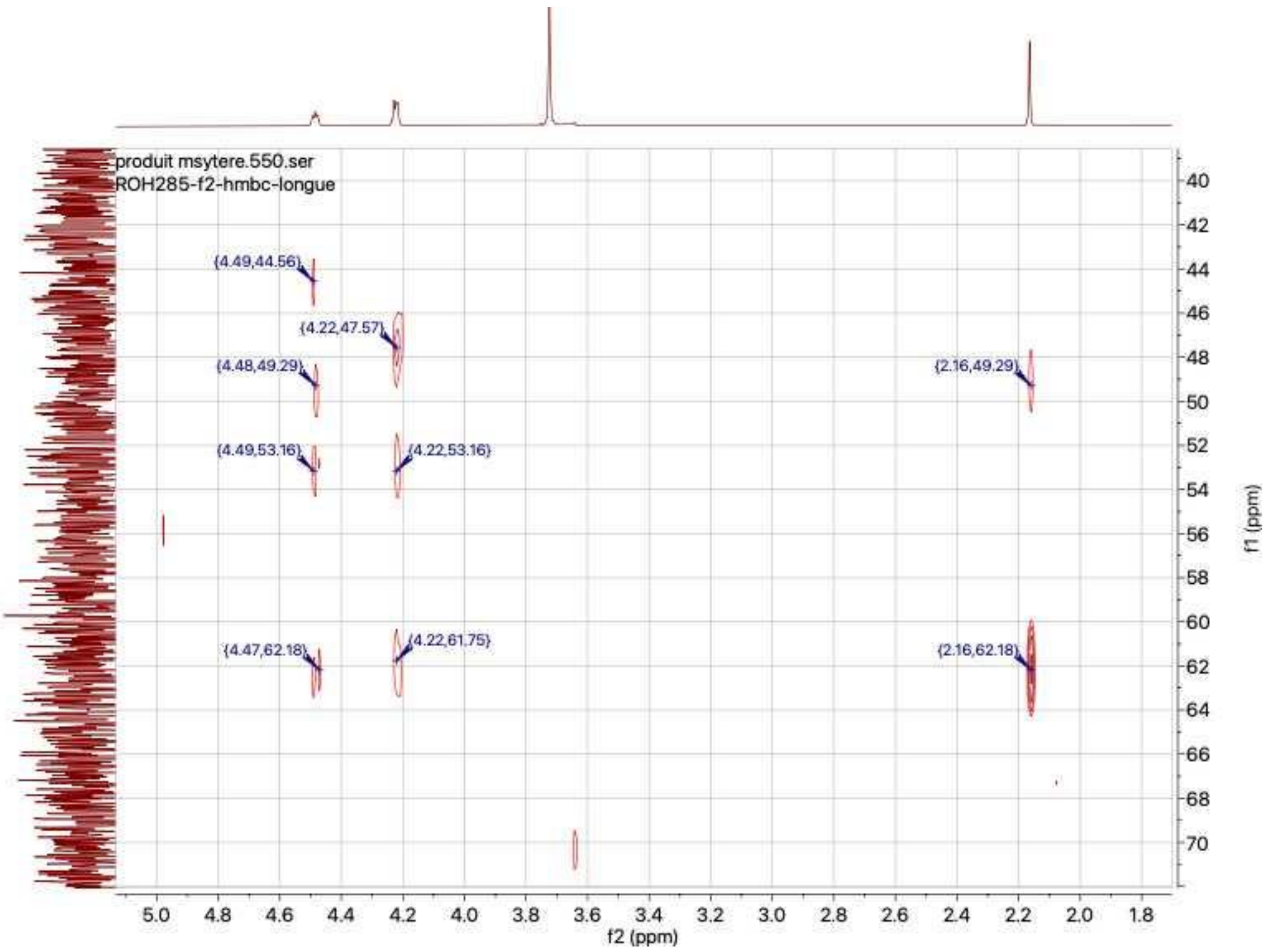

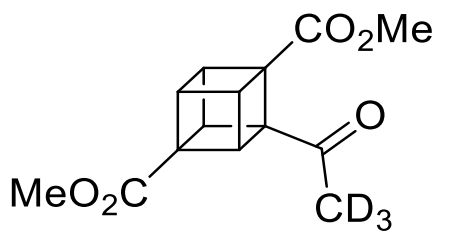

4-*d*$_3$, isolated in 5% yield (3 mg) when experiment was carried out in CD_3CN. **^{1}H NMR** (400 MHz, $CDCl_3$) δ 4.49 – 4.47 (m, 2H), 4.23 – 4.20 (m, 3H), 3.72 (s, 6H); **^{13}C NMR** (101 MHz, $CDCl_3$) δ 203.2, 171.0, 170.0, 62.3, 58.8, 53.3, 52.1, 52.0, 49.40 (2C), 47.80, 44.90 (2C), 29.50 (m). **HRMS** (ESI) *m/z:* [M+Na]$^+$ Calcd for $C_{14}H_{11}{}^2H_3O_5Na$ 288.0927; Found 288.0925.

^{1}H NMR (400 MHz, $CDCl_3$)

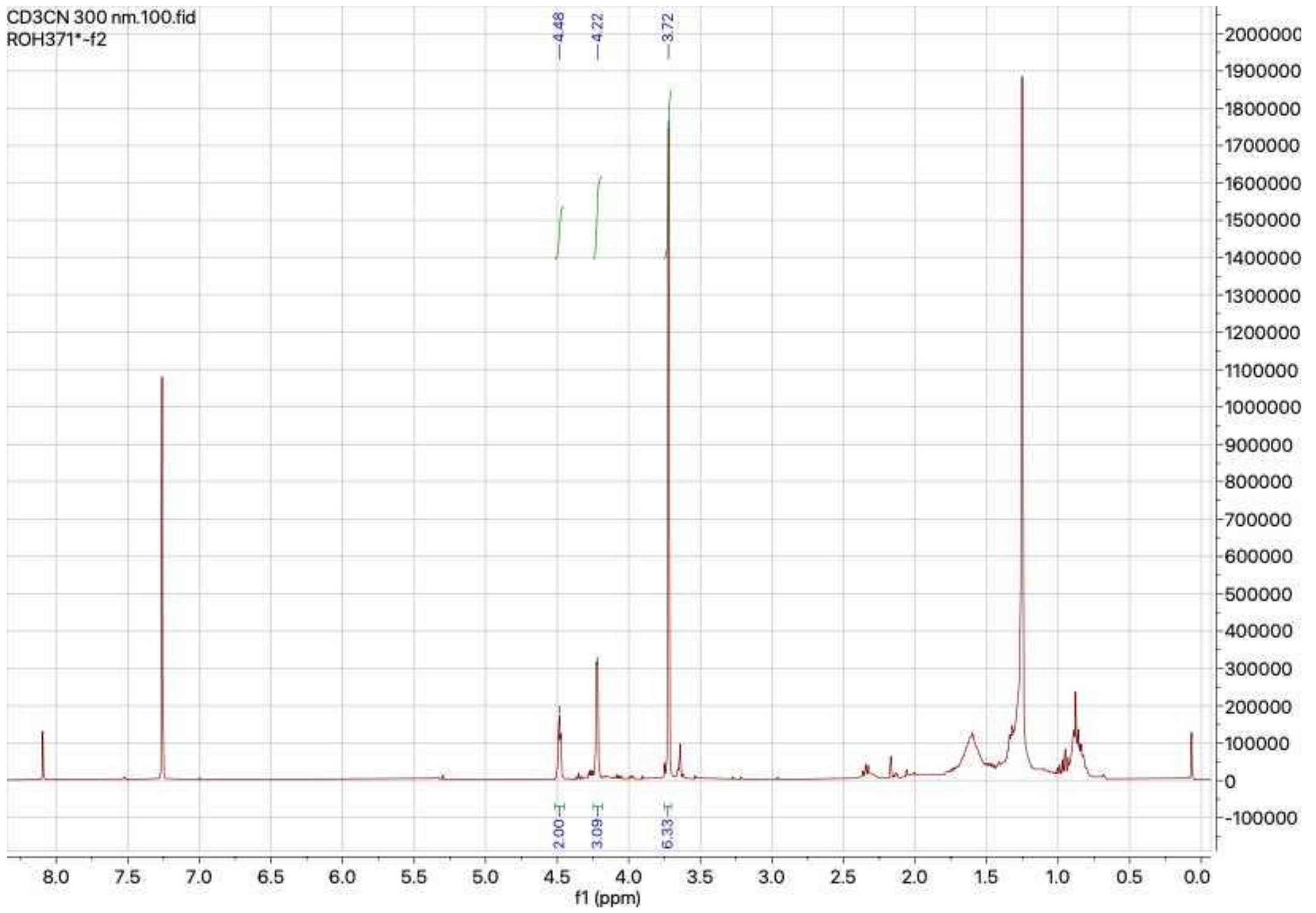

^{13}C NMR DEPT (101 MHz, $CDCl_3$)

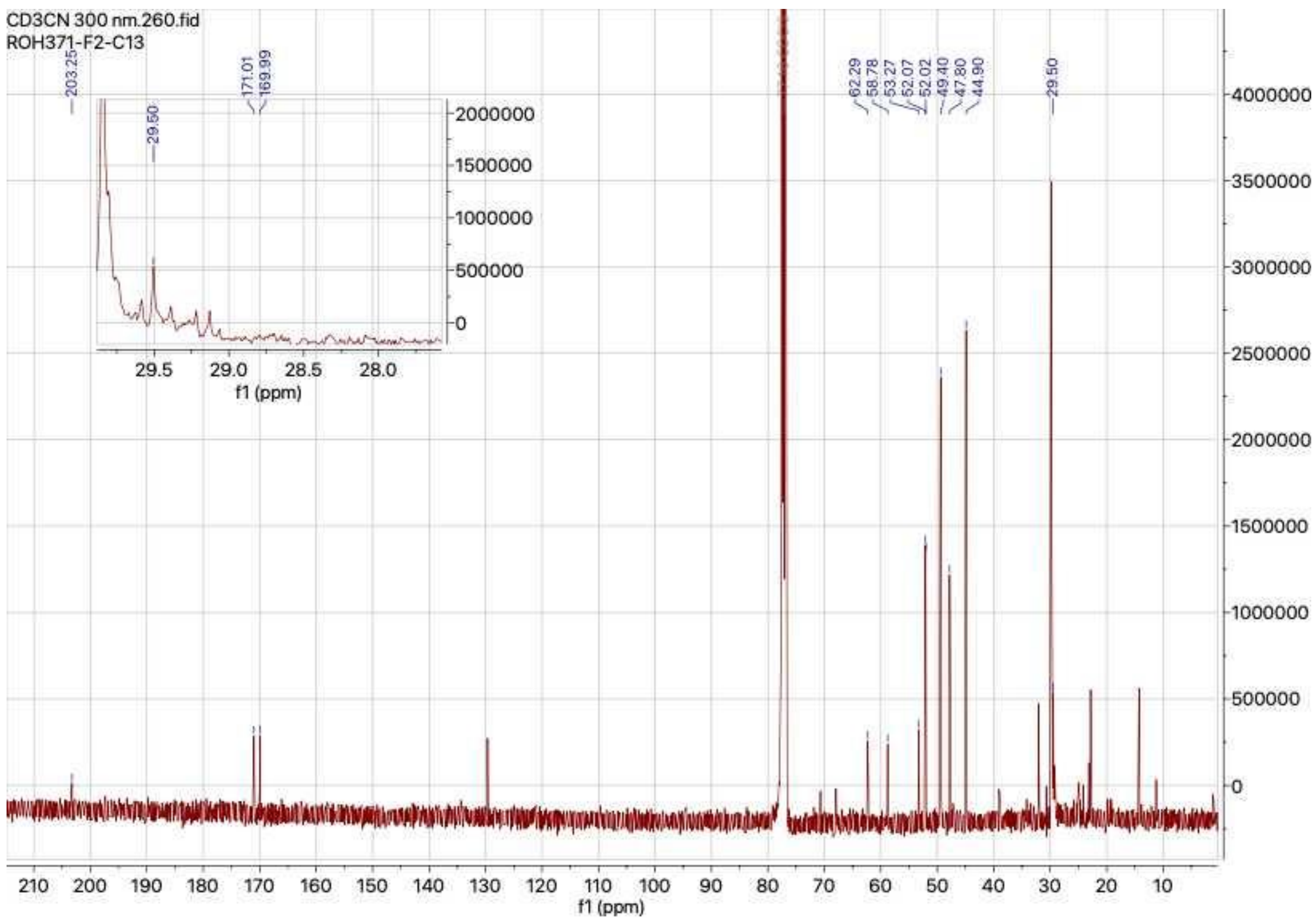

Synthesis of dimethyl 2-chlorocubane-1,4-dicarboxylate (3)

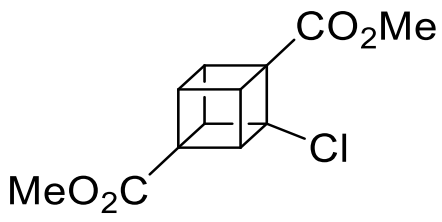

To a 4 mL disposable glass vial, equipped with a magnetic stir bar, was added dimethyl 1,4-cubanedicarboxylate (40 mg, 0.182 mmol, 1 equiv.) and 1,3-dichloro-5,5-dimethylhydantoin (DCDMH) (25 mg, 0.127 mmol, 0.7 equiv.). The vial was degassed with an argon flow and degassed acetonitrile (4 mL) was added. The solution was stirred and irradiated using the PhotoRedOx Box™ device from EvoluChem equipped with a EvoluChem LED 365PF during 4 h at a temperature between 25 and 40°C. Then, the reaction mixture was concentrated in vacuo. Purification by reverse phase chromatography (eluted with water/acetonitrile from 90/10 to 30/70) on a PF-15C18HP cartridge from Interchim afforded the desired product as a beige powder in 59% yield (27.5 mg).

mp 121°C; **^{1}H NMR** (300 MHz, $CDCl_3$) δ 4.45 – 4.33 (m, 2H), 4.32 – 4.16 (m, 3H), 3.76 (s, 3H), 3.72 (s, 3H); **^{13}C NMR** (75 MHz, $CDCl_3$) δ 170.5, 168.5, 69.6, 62.9, 56.3 (2C), 52.9, 52.1, 52.0, 48.1, 44.1 (2C); **IR** (AT, cm^{-1}): ν = 3006, 1715, 1437, 1327, 1218, 1106, 1091, 794; **HRMS** (API) *m/z:* $[M+H]^+$ Calcd for $C_{12}H_{12}O_4{}^{35}Cl$ 255.0424; Found 255.0422; *m/z:* $[M+H]^+$ Calcd for $C_{12}H_{12}O_4{}^{37}Cl$ 257.0395; Found 257.0401.

^{1}H NMR (300 MHz, $CDCl_3$)

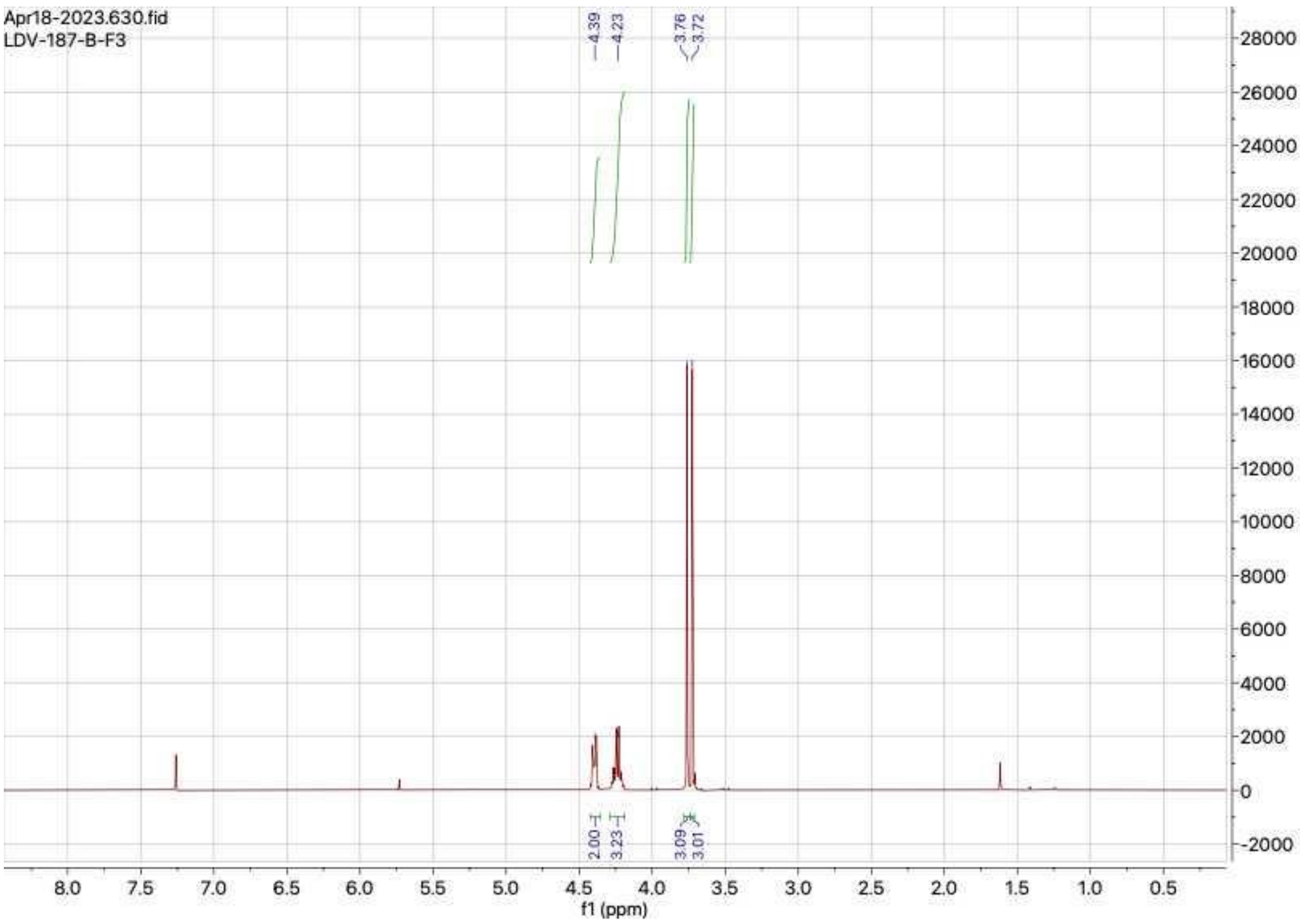

^{13}C NMR (75 MHz, $CDCl_3$)

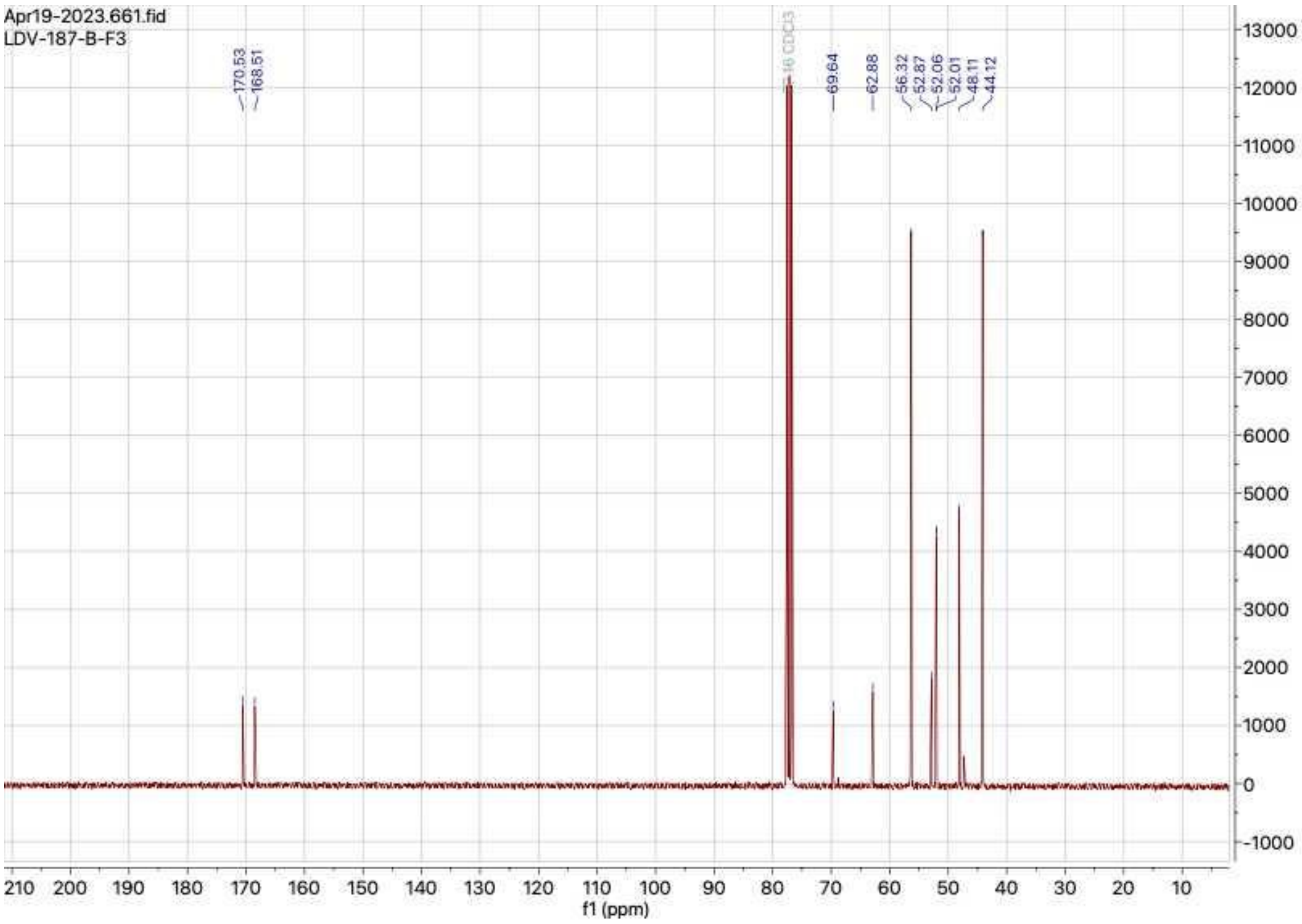

Chlorination in Flow

Dimethyl 1,4-cubanedicarboxylate **1** (120 mg, 0.545 mmol) and DCDMH (74.8 mg, 0,380 mmol, 0.7 equiv) were placed in a vial under argon atmosphere. Dry acetonitrile (6 mL, bottle) was added. The reaction mixture was stirred and passed through the photoflow reactor (310 nm - 28°C - residence time 30 min)

Then, the reaction mixture was diluted in CH_2Cl_2 and concentrated (4 mL collection: LDV-456-coeur + beginning and end of reaction: LDV-456-ext).

Purification of LDV-456-coeur was performed by reverse phase chromatography eluted with water/CH_3CN at 35% CH_3CN (LDV-456-B-F1, 26 mg of **1**) and at 40% CH_3CN (LDV-456-B-F2, 38 mg compound **3**).

Yield on 4 mL collection = 41%

Yield Based on Remaining Starting Material on 4 mL collection = 61%

^{1}H NMR spectra of the collected fractions "beginning and end of reaction" (2 mL) revealed a mixture of **1** and **3** (ratio ~4:1).

^{1}H NMR (300 MHz, $CDCl_3$) of the collected fractions “beginning and end of reaction”

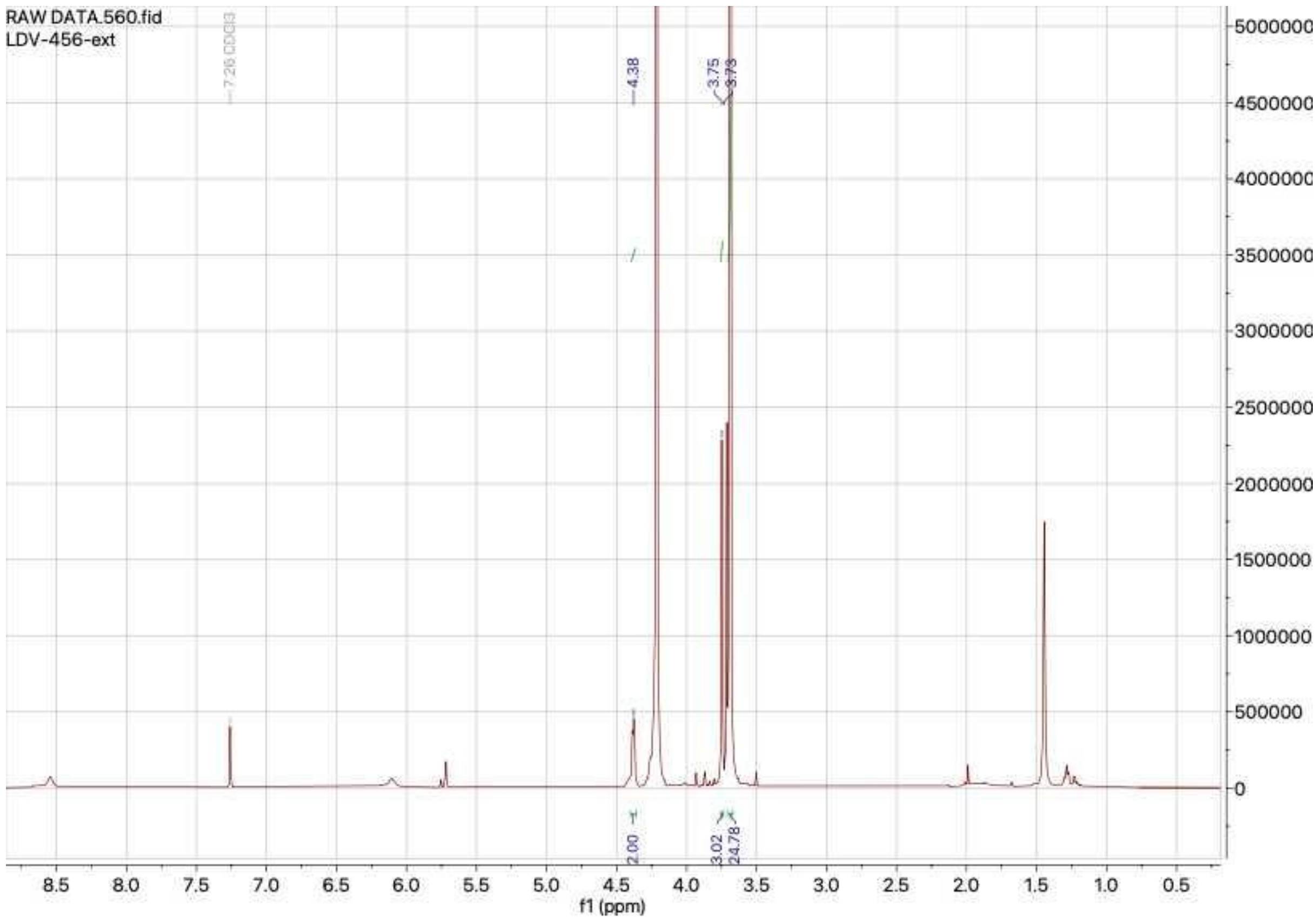

Supplement: Supplementary file 1 [file Beilstein_J_Org_Chem-22-1246-s001.pdf]
